# Supplementary material for: PhotoGate microscopy to track single molecules in crowded environments
Source: Nat Commun. 2017 Jan 10;8:13978. doi: 10.1038/ncomms13978 (PMC5234080; doi:10.1038/ncomms13978)
Supplement: Supplementary Information — Supplementary Figures and Supplementary Tables [file ncomms13978-s1.pdf]

Supplementary Figures:

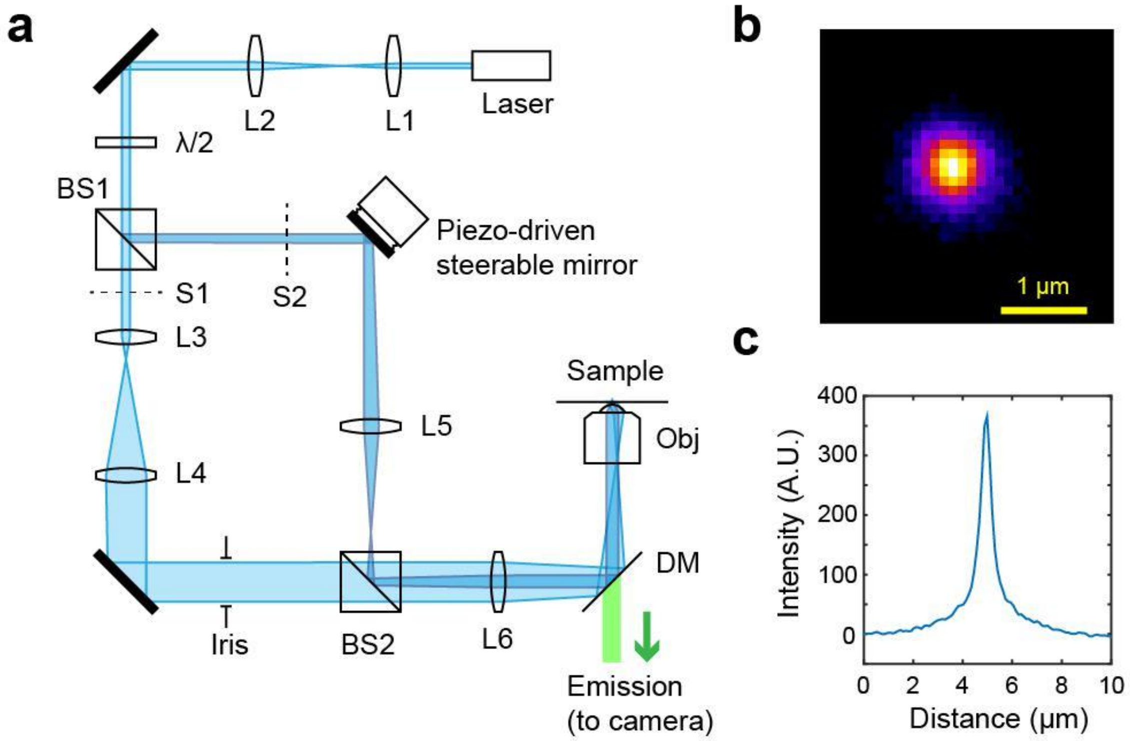

Supplementary Figure 1. PhotoGate Assay by Steering a Focused Laser Beam. (**a**) Optical diagram of the PhotoGate microscope. The beam exiting the laser is expanded to a ~3 mm diameter and collimated using a 3:1 telescope (lenses L1 and L2). The beam then passes through a half-wave plate (λ/2) mounted on a rotary stage, which allows us to control the relative amount of power going into the photogate and the TIRF beam. The polarized beamsplitter cube BS1 splits the beam into the TIRF and photogate components, which can be shuttered independently of each other by shutters S1 and S2. The TIRF beam is further expanded by a 5:1 telescope (L3 and L4) and focused onto the back-focal plane (BFP) of the objective by the TIRF lens L6. The diameter of the TIRF beam can be altered with a variable-diameter iris conjugate to the image plane. Meanwhile, the photogate beam is steered by the piezo-driven mirror and relayed onto the BFP of the objective by the 4:3 telescope made by lenses L5 and L6. Fluorescence emission from the sample passes through the dichroic mirror DM and is collected by an EMCCD camera. (**b**) Fluorescence image and (**c**) 1D intensity profile of the focused laser beam.

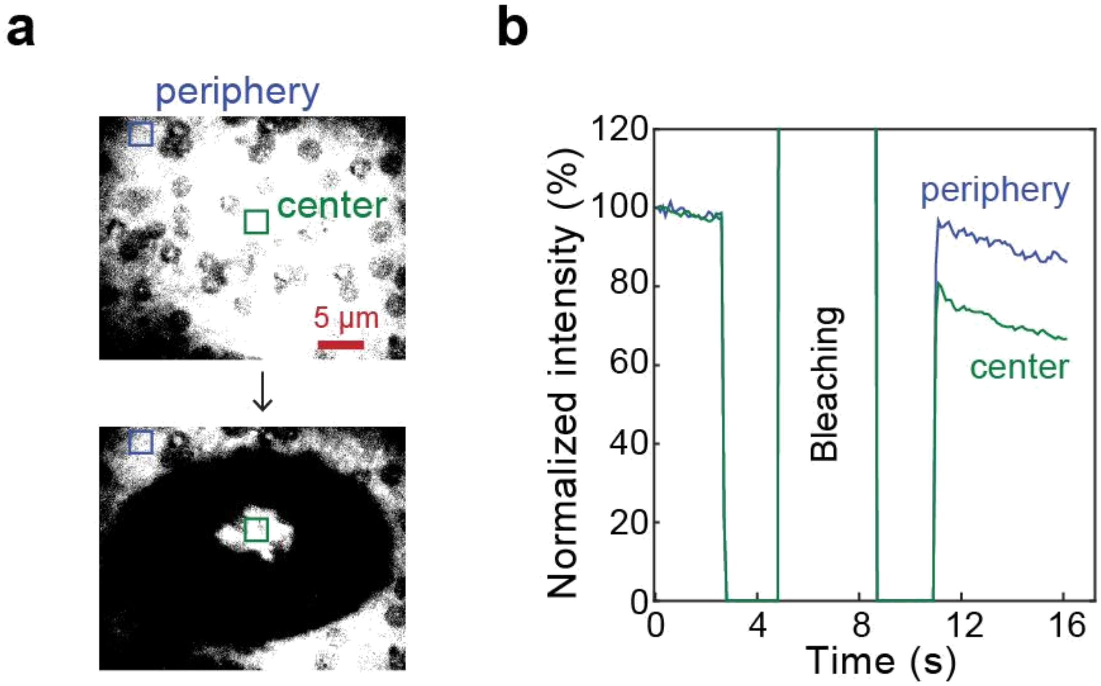

Supplementary Figure 2. Measurement of unwanted bleaching in the center of a donut-shaped ROI. (**a**) A coverslip densely coated with mEos2 is bleached in a donut-shaped pattern to measure the degree of unwanted bleaching in the center of the ROI (**b**) Normalized fluorescence intensities indicate that only 20% of the fluorescence signal in the center (red square in a) is lost following the aggressive photobleaching cycle. Photobleaching outside the periphery of the ROI (blue square in a) was negligible. Gradual reduction in fluorescence is due to photobleaching under TIRF illumination.

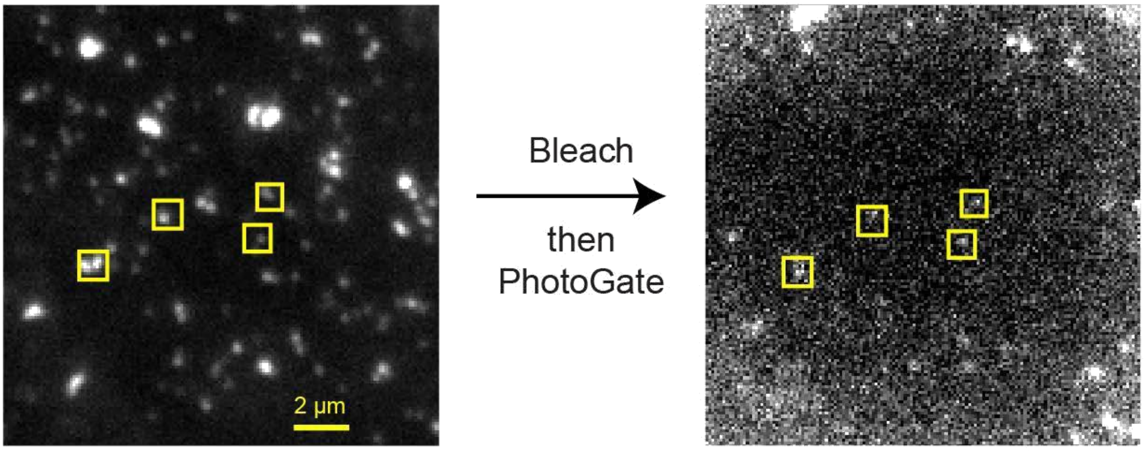

Supplementary Figure 3. Correlation between known endosome positions prior to bleaching and the appearance of single APPL1 spots in PhotoGate. (Left) Endosomes are relatively immobile in nocodazole-treated cells, and their positions (yellow squares) are determined prior to the PhotoGate assay. (Right) In PhotoGate assays, single APPL1 spots appear at previously known endosome locations.

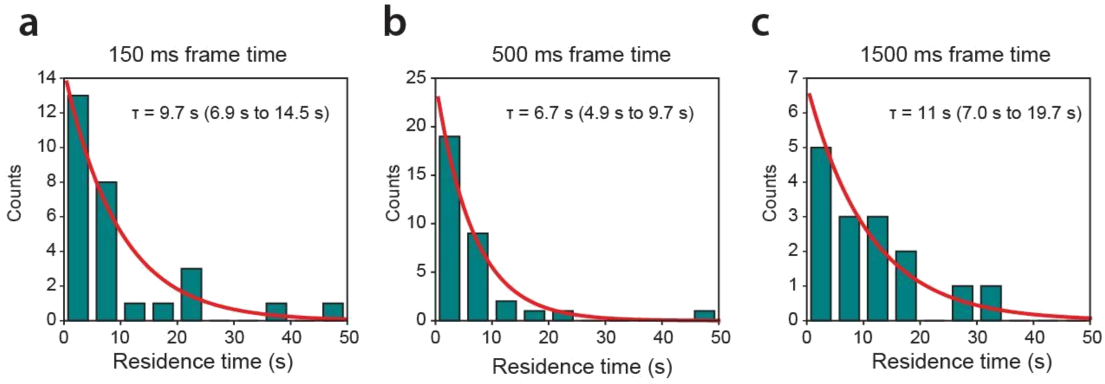

Supplementary Figure 4. Dissociation time constants of single APPL1 molecules from endosomes measured at different frame times. (**a-c**) Residence time histograms of single APPL1 molecules on endosomes at different frame times. The mean APPL1 off-rate (τ) is calculated by a single exponential decay (red curve, mean ± 95% conf. int.). The data were collected in time-lapse acquisition mode (**Methods**). In each data set, the exposure was fixed at 50 ms per frame, and the 'dark' time was altered to achieve the desired frame times (100 ms in **a**, 450 ms in **b**, and 1450 ms in **c**).

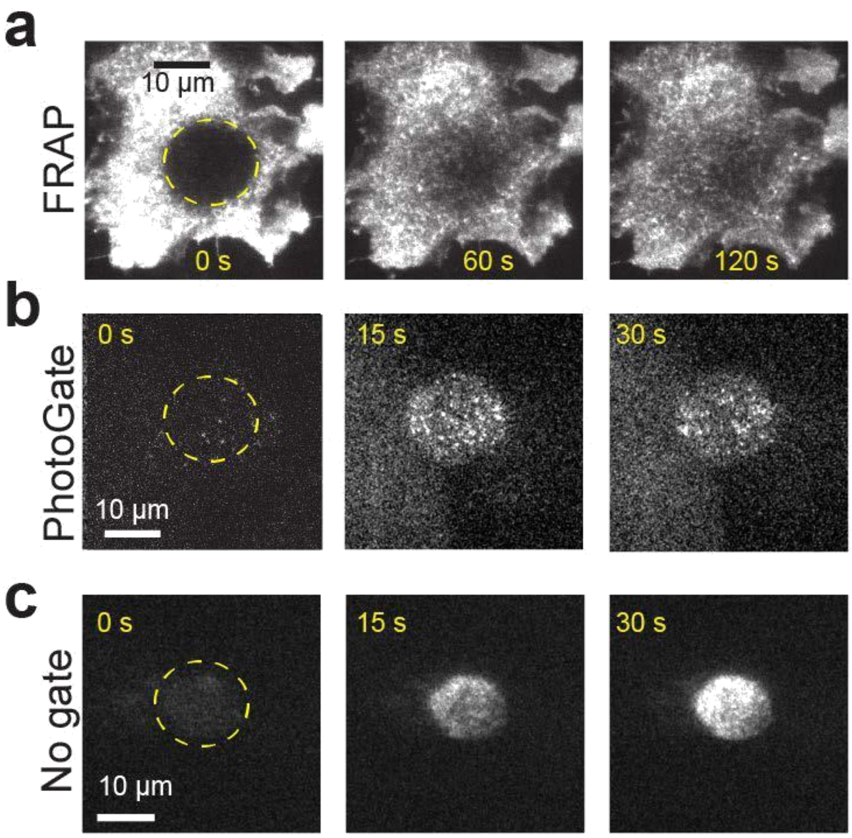

Supplementary Figure 5. Direct comparison of conventional FRAP, PhotoGate, and bleaching without subsequent gating, using an ROI of equal dimensions. (**a**) mNeonGreen-EGFR molecules exhibit a continuous recovery of fluorescent intensity in a FRAP assay (see **Supplementary Movie 6**). In this experiment, a 17 µm diameter area is bleached by sweeping the focused beam, then imaged continuously under whole-cell TIRF illumination with the focused beam shuttered. (**b**) In PhotoGate, when gating an ROI of equal diameter (17 µm) the effective density of single fluorophores remains nearly constant over 30 s (see **Supplementary Movie 2**). The focused beam is swept around the perimeter of the ROI every two seconds, and the TIRF beam is reduced in size in order to prevent unnecessary bleaching outside the ROI. (**c**) A 17 µm diameter area is bleached exactly as in a) and b), and imaged under reduced-diameter TIRF illumination as in b) but without periodically sweeping the focused beam around the perimeter. In the absence of gating, intensity quickly recovers past the single-molecule detection limit (see **Supplementary Movie 9**). The illuminated spot appears slightly smaller than in b) because the iris used to control the TIRF beam radius is manually controlled and imprecise.

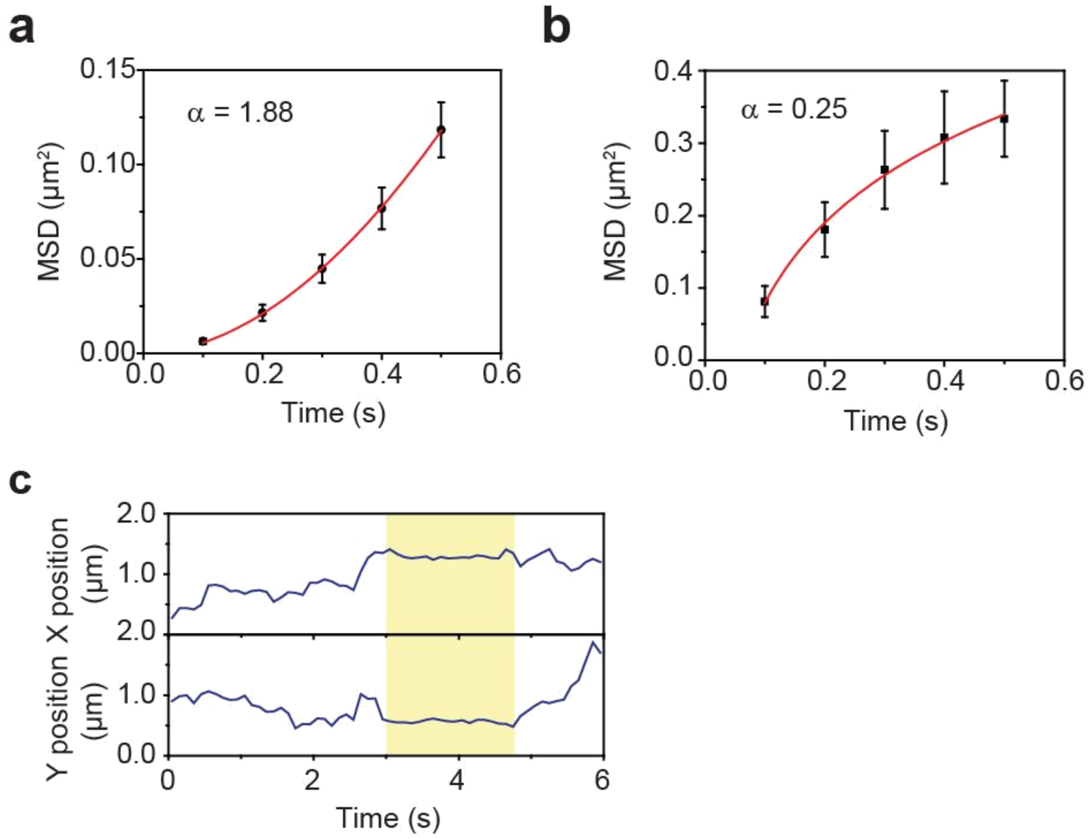

Supplementary Figure 6. MSD Plots of GFP-EGFR Spots that Display=2Non-

Diffusive+ Behavior and Anomalous Diffusion (**a**) The MSD plot was fitted to (red

0

curve). The value of the exponent (α) is close to 2, suggesting unidirectional transport of the molecule. (**b**) MSD plot of a single molecule shows that the variance increases sublinearly (α is smaller than 1) with time, suggesting confined diffusion of the molecule. (**c**) Diffusion of EGFR spots is interrupted by transient pauses (yellow shaded region). Transient pauses were observed in 3% of 156 spots for 1.3 ± 0.3 s (mean ± s.e.m.) in the presence of EGF, and 7% of 188 spots for 2.3 ± 0.8 s in the absence of EGF.

Supplementary Tables

Supplementary Table 1. Variable parameters used in computer simulations that were used to create figures.

FIGURE	5a	5a	5b	5b	5c	5c
Sub-panel	Gate	TOCCSL	Gate	TOCCSL	Gate	TOCCSL
diffusion_const	0.1	0.1	0.1	0.1	0.1	0.1
frame_rate	10	10	10	10	5	5
gauss_width	0.4	0.4	0.4	0.4	0.4	0.4
grid_delta	0.04	0.04	0.04	0.04	0.08	0.08
grid_num_el	1000	1000	1000	1000	500	500
intensity_tirf	2000	2000	20000	20000	0 to 13500	0 to 13500
num_time_steps	20000	20000	20000	20000	5000	5000
r_ring	8	8	8	8	8	8
r_tirf	7	7	7	100	7	7
steps_in_gate	25	25	25	25	6	6
steps_in_tirf	474	4999975	474	4999975	243	1249993
steps_per_frame	25	25	25	25	12	12
tau	0.004	0.004	0.004	0.004	0.016	0.016
track_min_distance	0.5	0.5	0.5	0.5	N/A	N/A
tracking_roi	7	7	7	7	N/A	N/A

FIGURE	5d	5d	5e	5e	5f	5f
Sub-panel	Gate	TOCCSL	0.1 um2/s	1um2/s	Gate	TOCCSL
diffusion_const	0.1	0.1	0.1	1	0.1	0.1
frame_rate	5	5	5	5	5	5
gauss_width	0.4	0.4	0.4	0.4	0.4	0.4
grid_delta	0.08	0.08	0.08	0.2529	0.08	0.08
grid_num_el	500	500	500	158	500	500
intensity_tirf	1500	1500	1000	1000	1000	1000
num_time_steps	5000	5000	5000	5000	5000	5000
r_ring	8	8	8	8	2 to 16	2 to 16
r_tirf	7	7	7	7	1 to 15	1 to 15
steps_in_gate	6	6	6	6	6	6
steps_in_tirf	243	1249993	618	53	243	1249993
steps_per_frame	12	12	12	12	12	12
tau	0.016	0.016	0.016	0.016	0.016	0.016
track_min_distance	N/A	N/A	N/A	N/A	N/A	N/A
tracking_roi	N/A	N/A	N/A	N/A	N/A	N/A

Supplementary Table 2. Variable parameters used in computer simulations that were used to create movies.

MOVIE	10	11	12	13
Sub-panel				
diffusion_const	0.1	0.1	0.5	0.5
frame_rate	10	10	10	10
gauss_width	0.4	0.4	0.4	0.4
grid_delta	0.08	0.08	0.089442719	0.089442719
grid_num_el	500	500	446	446
intensity_tirf	1000	1000	2000	2000
num_time_steps	5000	5000	20000	20000
r_ring	9	9	8	8
r_tirf	8	8	7	7
steps_in_gate	6	6	25	25
steps_in_tirf	118	124993	58	4999975
steps_per_frame	6	6	25	25
tau	0.016	0.016	0.004	0.004
track_min_distance	N/A	N/A	0.5	0.5
tracking_roi	N/A	N/A	7	7

Supplementary Table 3. Parameters common to all computer simulations.

Parameters identical for all simulations:	
box_width	40
grid_size	40
intensity_gate	3000000
num_particles	80000
num_pre_bleach_cycles	50
num_steps	50
oligo_state	2
photon_budget	50000
sim_time	80
